# Effect of virtual reality on perioperative anxiety, stress and pain in total hip arthroplasty: a randomized controlled trial

**DOI:** 10.1186/s12871-025-03316-3

**Published:** 2025-09-09

**Authors:** Saad Ahmed Moharam, Mohammed Said ElSharkawy, Ahmed Mohamed ELkashef, Mohamed Abdelhamed Romeih, Ahmed Osama Abd Rab El Rasool, Mostafa Mohamed Shaheen

**Affiliations:** 1 Faculty of Medicine, Anesthesiology, Surgical Intensive Care and Pain Medicine Department, Tanta University, Tanta, El Gharbia, 31511 Egypt; 2 Faculty of Medicine, Orthopedic Surgery Department, Faculty of Medicine, Tanta University, Tanta, Egypt; 3 Faculty of Medicine, Neuropsychiatry Department, Tanta University, Tanta, Egypt

**Keywords:** Virtual reality, Anxiety, Stress, Pain, Total hip arthroplasty

## Abstract

**Background:**

Virtual reality (VR) has shown promise as a nonpharmacological alternative to pharmaceutical pain relievers and anxiety medications in clinical trials by decreasing pain and anxiety in orthopedic surgeries. The aim of the study was to evaluate the impact of VR on these outcomes in individuals undergoing total hip arthroplasty (THA).

**Methods:**

This randomized, controlled, open-label research included 50 participants planned for THA with spinal anesthesia (SA). Patients were allocated equally to group VR: patients were immersed in a peaceful natural environment with soft music preoperatively and intraoperatively, and group C did not receive VR.

**Results:**

The STAI-S for anxiety and PSS-10 scores for stress were significantly lower in group VR before SA and immediately postoperatively (*P* < 0.05). Hemodynamics at 5 min, 30 min, and 60 min, pain scores at 4 h and 6 h, 24 h pethidine consumption, haloperidol dose, and cortisol level at 6 h postoperative were decrease in group VR in comparision to group C (*P* < 0.05). Time to first analgesia request and satisfaction level were higher in group VR in comparision to group C (*P* < 0.05).

**Conclusions:**

VR can reduce perioperative anxiety, stress, pain, and opioid requirements, and improve satisfaction in THA patients.

**Trial registration:**

The trial was registered https://clinicaltrials.gov/study/NCT06088069?id=NCT06088069&rank=1((ID:NCT06088069, Principal investigator: (SAAD AHMED MOHARAM, Date of registration: 18-10-2023).

## Background

The term “virtual reality” (VR) refers to a type of computer-generated technology that creates an immersive experience by simulating a three-dimensional environment and facilitating the user’s interaction with it. The cost of VR technology has decreased and accessible in recent years. Also, it has gained increasing interest from researchers and clinicians for applications in various healthcare domains, such as neuroscience, physical therapy, psychology, rehabilitation, and surgery [1–3]. Several randomized controlled trials have reported the feasibility of using VR following total hip arthroplasty (THA) for rehabilitation [4–7].

For individuals with advanced hip osteoarthritis, THA is the gold-standard therapeutic option. This surgical treatment has a solid track record of success and is performed often around the world [8, 9]. THA surgery is commonly characterized by moderate to severe postoperative pain [10].

Anxiety in patients undergoing THA could cause physiological changes like tachycardia and hypertension, which can force the surgeon to postpone the surgery or bother the surgeon intraoperatively [11, 12]. Additionally, a high degree of postoperative analgesic demand, delayed wound healing, extended hospital stays, elevated risk of infection, and greater expenses are all possible outcomes of perioperative anxiety and stress [13, 14].

VR has shown promise as a nonpharmacological alternative to pharmaceutical pain relievers and anxiety medications in clinical trials by decreasing pain and anxiety in orthopedic surgeries [15, 16], total knee arthroplasty (TKA) [14, 17, 18], ambulatory surgery [19], septorhinoplasty [2], and THA [4].

Even though previous research has examined the role of VR in postoperative rehabilitation after THA, the current research intended to assess the impact of VR on perioperative anxiety and stress and pain in patients who are undergoing THA.

## Methods

This open-label, controlled trial with random design was conducted on 50 participants of age 21 or older, both sexes, with a physical status of I-III as defined by the American Society of Anesthesiologists (ASA), listed for elective THA performed under spinal anesthesia (SA) from 19th October 2023 till 5th August 2024.

This research was approved by the Institutional Ethical Committee, Faculty of Medicine, Tanta University, Tanta, Egypt, (Approval code: 36264PR331\9\23). The trial was registered https://clinicaltrials.gov/study/NCT06088069?id=NCT06088069&rank=1 (ID: NCT06088069), Principal investigator: (SAAD AHMED MOHARAM, Date of registration: 18-10-2023). This study was conducted in compliance with the Helsinki Declaration. All subjects who participated in the trial given written, informed consent. Prior to patient enrollment.

Patients were excluded if they had adrenal insufficiency, blindness, deafness, cerebrovascular disease, chronic sedative or narcotic use, substance addiction, claustrophobia, psychiatric-cognitive dysfunction, and uncooperative behavior.

### Randomization

The patients were allocated randomly using computer-generated randomization numbers parallelly to two equal groups: Group VR and Group C (control group), which did not receive any VR experience. Due to the difference in technique, the study was conducted in an open-label manner.

Preoperatively, a detailed history was taken, a clinical examination was performed, and routine laboratory investigations were conducted.

All patients underwent spinal anesthesia performed under strict aseptic conditions. A 25G Quincke spinal needle was inserted at the L3–L4 interspace, and 2.5 mL of 0.5% hyperbaric bupivacaine was administered intrathecally. No intrathecal adjuvants (such as fentanyl or morphine) were used to ensure standardization of the anesthetic technique across the study groups.

The State Anxiety Inventory (STAI-S) for anxiety was demonstrated to patients, the Perceived Stress Scale (PSS-10) for stress, and the Numerical Rating Scale (NRS) for pain. The paramedical team cordially welcomed and made necessary arrangements for patients participating in the trial to guarantee a seamless ambulant medical encounter.

In the VR group, patients were enveloped in a serene environment with nature scenes and audio features of soft music, completely isolating them from the actual world for a period of 15 min before surgery and during the procedure using VR glasses with an audio headset.

Heart rate (HR) and mean arterial blood pressure (MAP) assessed at baseline, before SA, and at 5, 10, 15, 30, 60, 90, and 120 min during the surgery, as well as at the end of surgery.

The Arabic-validated versions of the STAI-S [20] and PSS-10 [21] were measured at three different stages: 15 min before the surgery (baseline), before SA, and immediately postoperative.

Intraoperatively, SA was carried out under complete aseptic conditions. If the patient showed signs of anxiety, haloperidol was administered (2.5 mg at gradually increasing doses until the target effect was reached), and total haloperidol consumption was documented.

Postoperatively, the NRS was recorded 30 min, 2, 4, 6, 12, 18, and 24 h following the operation. All patients were given routine analgesia as 1 g of paracetamol IV. If their NRS score exceeded three, they received rescue analgesia with IV pethidine (0.5 mg/kg). The total amount of pethidine consumed within the first 24 h post-surgery and the time to the first dose of rescue analgesia were documented.

The levels of serum cortisol were assessed before and six hours following the surgical procedure. The level of patient satisfaction was evaluated using a 3-point scale (1 = unsatisfied, 2 = neutral, 3 = satisfied) after discharge to the ward.

Anxiety was the primary outcome of the research. Included in the secondary outcomes were hemodynamic parameters, stress, pain score, the cumulative opioid dose administered within the initial twenty-four hours following the operation, total haloperidol dose during the operation, serum cortisol, and patient satisfaction.

### Sample size calculation

Sample size calculations were conducted using G*Power 3.1.9.2 (Universitat Kiel, Germany). A pilot study that was not published was conducted, with each category contains five cases., and we found that the mean (± standard deviation (SD)) of STAI score before SA (the primary outcome) was 36.2 ± 7.8 in group VR and 44.2 ± 9.67 in group C. The total number of samples was determined by the following: effect size of 0.906, the study’s power is 80%, and the confidence level is 95%, the group ratio is 1:1, and The inclusion of four cases in each cohort was necessary to overcome drop-out. Consequently, we recruited 25 patients for each group.

### Statistical analysis

Utilizing SPSS v27 (IBM^©^, Armonk, NY, USA), statistical analysis was carried out. To check if the data was distributed normally, we used histograms and the Shapiro-Wilks test. The unpaired Student’s t-test was employed to evaluate the quantitative parametric data, which were presented as mean and SD. The median and interquartile range (IQR) were used to assess quantitative non-parametric data, which were expressed as the Mann-Whitney test method. The qualitative variables, which were expressed as frequency and percentage, were analyzed using either the Chi-square test or Fisher’s exact test, as appropriate. A two-tailed *P* value that was less than or equal 0.05 was considered statistically significant.

## Results

**Fig. 1:**
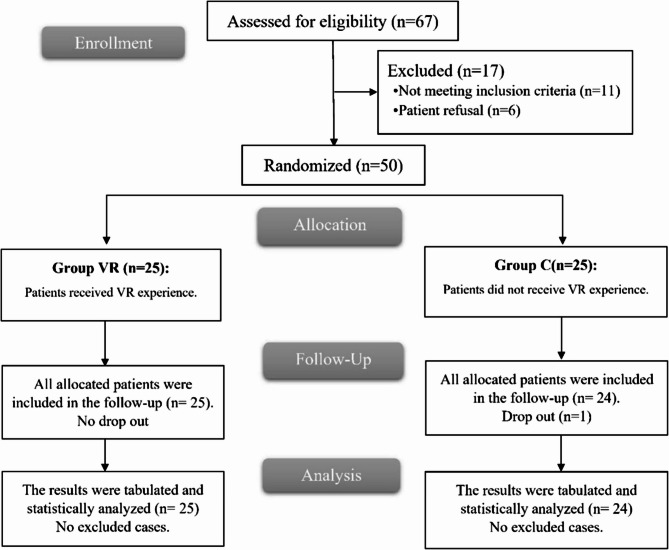
CONSORT flowchart of the enrolled patients

Out of 67 patients who were evaluated for eligibility, 6 declined to participate, and 11 did not fulfill the requirements. Two equal groups of 25 patients in each one using a random allocation method. The statistical analysis was performed on all assigned patients in group VR and 24 patients in group C (one patient dropped out due to the need for intraoperative and postoperative mechanical ventilation). Figure 1.

In both categories, the patient’s characteristics, comorbidities, and the duration of the surgery were comparable. Table 1.

**Table 1:** Patient’s characteristics, comorbidities and duration of surgery of the studied groups

		Group VR (*n* = 25)	Group C (*n* = 24)	*P*
Age (years)		53.76 ± 10.03	52.79 ± 11.87	0.759
Sex	Male	10 (40%)	13 (54.17%)	0.321
	Female	15 (60%)	11 (45.83%)	
Weight (kg)		83.28 ± 9.8	82.71 ± 13.48	0.865
Height (cm)		166.76 ± 6.74	167.13 ± 6.32	0.846
BMI (kg/m ^2^ )		29.98 ± 3.48	29.66 ± 4.95	0.795
ASA physical status	I	9 (36%)	6 (25%)	0.693
	II	12 (48%)	13 (54.17%)	
	III	4 (16%)	5 (20.83%)	
Comorbidities	Diabetes mellitus	7 (28%)	5 (20.83%)	0.741
	Hypertension	9 (36%)	6 (25%)	0.404
	Hypothyroidism	2 (8%)	4 (16.67%)	0.417
Duration of surgery (min)		86.8 ± 21.35	91.25 ± 23.56	0.492

Data are presented as mean ± SD or frequency (%)

*BMI* body mass index, *ASA* American Society of Anesthesiologists

**Fig. 2:**
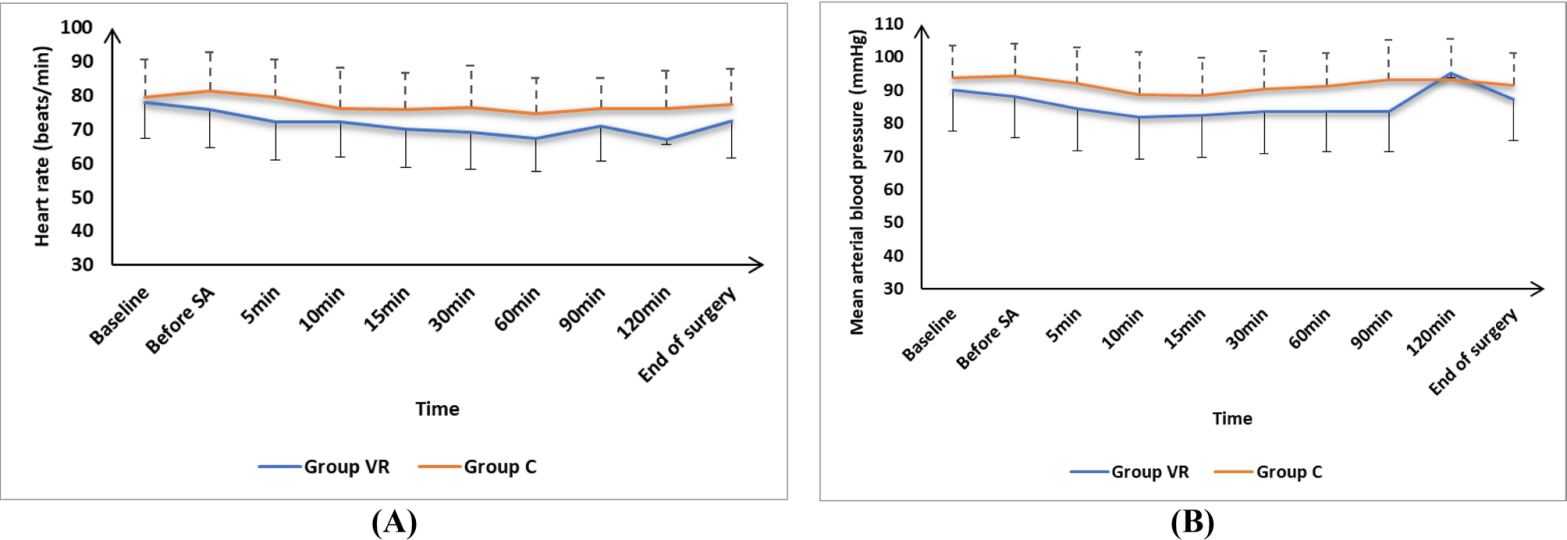
(**A**) Heart rate (HR) and (**B**) mean arterial blood pressure (MAP) changes of the studied groups

HR and MAP measurements at baseline, before SA, 10 min, 15 min, 90 min, 120 min, and the end of surgery were not substantially different between the two categories and were showed significante reduced at 5 min, 30 min, and 60 min in group VR in contrast to group C (*P* < 0.05). Figure 2.

At baseline, the STAI and PSS-10 scores results were comparable between the two categories. However, group VR exhibited notably lower STAI (*P* = 0.003) and PSS-10 (*P* < 0.001) scores compared to group C before SA. Immediately postoperatively, group VR also had significantly lower STAI (*P* = 0.032) and PSS-10 (*P* < 0.001) scores compared to group C, indicating reduced anxiety and PSS-10.

Regarding pain assessment using the NRS, slight differences were observed between both groups at 30 min, 2, 12, 18, and 24 h postoperatively. However, group VR experienced markedly decreased pain scores at 4, and 6 h (*P* < 0.001 and 0.021, respectively) postoperatively in contrast to group C, suggesting better pain management in the early postoperative period with the use of VR. Table 2.

**Table 2:** STAI-S, PSS-10, and NRS scores of the studied groups

		Group VR (*n* = 25)	Group C (*n* = 24)	*P*
STAI	Baseline	42.52 ± 7.83	43.13 ± 8.87	0.801
	Before SA	38.32 ± 7.89	45.63 ± 8.72	0.003
	Immediately postoperative	32.88 ± 7.77	38.13 ± 8.85	0.032
PSS-10	Baseline	25.72 ± 5.41	23.54 ± 3.72	0.109
	Before SA	21.32 ± 5.6	27.54 ± 3.67	< 0.001
	Immediately postoperative	16.8 ± 5.55	21.83 ± 3.61	< 0.001
NRS	30 min	0(0–0)	0(0–0)	---
	2 h	0(0–0)	0(0–0)	---
	4 h	2(2–3)	3(2–4.25)	< 0.001
	6 h	2(1–4)	2.5(2–6)	0.017
	12 h	3(2–5)	4.5(3–5)	0.076
	18 h	4(3–5)	5(3–6)	0.326
	24 h	4(3–5)	4(3–6)	0.205

Data are presented as mean ± SD or median (IQR)

*STAI* State-Trait Anxiety Inventory, *SA* Scheduled for elective THA under spinal anesthesia, *PSS-10* Perceived stress scale, *NRS* Numerical rating scale

Patients in group VR showed significant delay in time to the first request for rescue analgesia (6.04 ± 1.06 h) compared to group C (5.04 ± 0.91 h) (*P* < 0.001). Additionally, the total amount of pethidine consumed within the initial 24 h, patients required haloperidol, total haloperidol consumption, and opioid requirements were substantial reduced in group VR in contrast to group C (*P* < 0.05).

Serum cortisol levels, a marker of physiological stress, were similar before surgery but significantly lower in group VR at 6 h postoperatively compared to group C (*P* = 0.006). Finally, Compared to group C, patient satisfaction levels were markedly more elevated in group VR. (*P* = 0.019). Table 3.

**Table 3:** Time to first request of rescue analgesia, pethidine consumed, haloperidol dose, serum cortisol level, and patient satisfaction of the studied groups

		Group VR (*n* = 25)	Group C (*n* = 24)	*P*
Time to first request of rescue analgesia (h)		6.04 ± 1.06	5.04 ± 0.91	< 0.001
Total dose of pethidine consumption in the first 24 h (mg)		112 ± 26.93	151.67 ± 52.47	0.002
Patients required haloperidol		7 (28%)	22 (91.67%)	< 0.001
Total haloperidol consumption (mg)		4.29 ± 2.38	8.18 ± 1.92	< 0.001
Serum cortisol level	Before surgery	28.12 ± 7.72	31.04 ± 5.83	0.143
	6 h postoperative	19.84 ± 5.66	24.75 ± 6.18	0.006
Patient Satisfaction	Satisfied	18 (72%)	9 (37.5%)	0.019
	Neutral	7 (28%)	11 (44%)	
	Unsatisfied	0 (0%)	4 (16.67%)	

Data are presented as mean ± SD or frequency (%)

## Discussion

The VR technology has emerged as a promising technique during preoperative [2, 22, 23], intraoperative [24, 25], and postoperative [4, 15, 16] setting, offering potential benefits in managing anxiety, stress, and pain.

Elevated levels of catecholamines, which are associated with perioperative anxiety and stress, can lead to an increase in both MAP and HR, potentially resulting in the development of arrhythmia [26]. There were notable reductions at 5 min, 30 min, and 60 min in HR and MAP in the VR group in contrast to group C. The findings of this research were similar to prior findings conducted by Baytar and Bollucuoğlu [2] who reported significant decreases in physiological outcomes, including MAP and HR, in patients undergoing septorhinoplasty when VR interventions were employed during the perioperative period, specifically before anesthesia and at 5, 10, and 15 min during VR exposure. Also, similar findings were notcied with Güneş and Sarıtaş [14] who reported that VR reduces perioperative HR in patients who undergoing TKA. Furthermore, Ontañón et al. [27] demonstrated that HR and MAP were notably decreased in the VR group in contrast to the no-VR group.

However, these findings disagree with Tashjian et al. [28] who reported slight HR changes between pre-VR and post-VR assessments in hospitalized pain patients. This difference can be attributed to extensive rehabilitation incorporated in their study and the relaxing effect of soft music where they used motivational music.

Anxiety before surgery increases the risk of physical and mental health issues, as well as behavioral and surgical difficulties [19]. Our study demonstrated VR’s efficacy in decreasing preoperative anxiety and stress levels as well. Compared to the control group, the group VR had markedly reduced STAI and PSS-10 scores before SA and immediately postoperatively. Also, the haloperidol dose was notably reduced in group VR in comparison to group C. These consistent results across studies highlight VR’s potential as an effective tool for managing preoperative anxiety and stress. For instance, Baytar and Bollucuoğlu [2] found that VR significantly reduced preoperative anxiety (STAI scores) in patients undergoing septorhinoplasty. This was in agreement with Ontañón et al. [27] who reported that During oral surgery, VR spectacles were effective in alleviating patient anxiety. Similarly, Carella et al. [24] and Öz et al. [29] demonstrated that VR effectively reduced anxiety levels in knee arthroplasty surgery and gynecological procedures respectively. In addition, Lier et al. [30] showed markedly reduced daily stress and anxiety levels in VR intervention groups contrasted with the control group. Also, Eijlers et al. [22] conducted a systematic review and meta-analysis, and it was determined that VR could alleviate some of the anxiety that children experience during surgeries. The lower anxiety and perceived stress in the group VR because of VR help patients shift their focus away from the surgical procedure and associated worries. As demonstrated by Benchimol-Elkaim et al. [23], Children’s perioperative anxiety can be alleviated with VR natural meditation.

Serum cortisol levels, a marker of physiological stress, were similar between the groups before surgery, although by 6 h after surgery, the group VR group had considerably reduced compared to group C.

Similarly, Ganry et al. [19] found that VR use led to reduced levels of salivary cortisol, a marker of stress, in patients experiencing preoperative anxiety before maxillofacial surgeries. Also, Nambi et al. [31] stated a substantial decrease in serum stress hormones as well as serum cortisol in patients who suffer from chronic low back pain who received VR therapy. This reduction in cortisol levels indicates that VR not only helps in managing stress but also has a measurable impact on physiological stress markers.

Regarding pain assessment using the NRS, there was a marked reduction in pain scores in the group VR at 4, and 6 h following the operation compared to group C, which proposes that VR could improve pain management during the first few hours after surgery. This reduction in opioid requirements enhances patient outcomes while decreasing the likelihood of opioid-related side effects. Compared to group C, patients in the group VR required (6.04 ± 1.06) hours before requesting rescue analgesia, whereas the control group only required (5 ± 0.91) hours (*P* < 0.001). Furthermore, the total amount of pethidine consumed within the initial 24 h was notably decreased in group VR than group C, indicating better pain control with VR intervention.

Several research have investigated the potential of VR to alleviate postoperative pain, with mixed results. Fuchs et al. [17] reported that early VR intervention following primary total knee arthroplasty significantly reduced pain and improved function but without effect on the long term. Moreover, Cohen et al. [32] and Öz et al. [29] who demonstrated that compared to control, VR led to lesser procedure-related pain scores. In contrast, Gianola et al. [18] found that early VR rehabilitation had no discernible impact on pain levels among individuals after total knee arthroplasty. Also, Araujo-Duran et al. [4] found that VR distraction did not effectively reduce acute pain after hip arthroplasty.

The ability of VR to modulate pain perception can be attributed to its capacity to engage multiple sensory modalities, thereby diverting attention from nociceptive stimuli as reported by Combalia et al. [15]. This is corroborated by findings from McSherry et al. et al. [33] who observed total opioid administration during VR therapy markedly decreased the amount of pain experienced by adults during unpleasant wound care procedures compared to when no VR was employed. Similarly, Smith et al. [34] performed a systematic review and discovered that VR can successfully manage acute pain and reduce opioid use in medical inpatients.

Finally, patient satisfaction levels were significantly higher in group VR, with 72% of patients reporting being satisfied, compared to only 36% in group C. This aligns with studies by Ehioghae et al. [16] who confirmed that patients who underwent orthopedic surgery have a great deal to gain from the perspective of VR-based therapy both postoperative healing and patient happiness. Similarly, Bernaerts et al. [35] reported that after utilizing relaxation-VR, pediatric patients reported considerably increased levels of happiness compared to baseline. In addition, Öz et al. [29] reported that compared to the control group, The VR group demonstrated a substantially higher level of patient satisfaction. The results of these trial indicate that the promise of VR in improving perioperative treatment and the patient experience as a whole.

This study has many limitations, including limited sample size. potential bias due to its open-label design, inability to blind participants and researchers because of the intervention’s noticeable effects or reliance on self-reported experiences, and limited external validity due to being conducted at a single center.

## Conclusions

VR can reduce perioperative anxiety, stress, pain, and opioid requirements and improve satisfaction in THA patients. VR is a promising technique for enhancing recovery after surgery.

## Data Availability

Data is available on reasonable requests from corresponding author.

## Abbreviations

ASA: American Society of Anesthesiologists
HR: Heart rate
IQR: Interquartile range
MAP: Mean arterial blood pressure
NRS: Numerical rating scale
PSS-10: Perceived stress scale
SA: Spinal anesthesia
SD: Standard deviation
STAI-S: State anxiety inventory
THA: Total hip arthroplasty
TKA: Total knee arthroplasty
VR: Virtual reality
